# Mucosal Coverage of Palatine Spines: CT Morphometric Analysis and Clinical Relevance

**DOI:** 10.7759/cureus.89766

**Published:** 2025-08-10

**Authors:** Layth R Alkhani, Yusef Qazi, Tarik F Massoud

**Affiliations:** 1 Engineering, Stanford University, Palo Alto, USA; 2 Computer Science, Stanford University, Palo Alto, USA; 3 Radiology, Stanford University School of Medicine, Palo Alto, USA

**Keywords:** dental implants, maxilla, mucous membrane, palate hard, torus palatinus

## Abstract

Introduction: The palatine spines (PSs) form the margins of the palatal grooves (PGs) of the hard palate, which contain the greater palatine arteries and nerves. Except for one overly simplistic account in the early 1990s, there are no detailed descriptions and dimensions for the mucosa covering the PSs and grooves.

Methods: We retrospectively studied the normative morphometric features of PSs and their overlying mucosa in selected 69 patients using CT imaging. We statistically analyzed variables among the demographics, morphometrics, and several other spine characteristics, with statistical significance set at p < 0.05.

Results: Lengths of mucosal coverage for bilateral spines varied from 3.5 ± 0.9 mm to 3.7 ± 1.1 mm. There was a statistically significant sex difference for the right middle spine size, larger in men (p = 0.044). The right middle and left medial spine sizes were significantly correlated with their mucosal coverage (p = 0.003 and p = 0.021, respectively). There was also a positive correlation between the right medial spine mucosal coverage and patient age (p = 0.001).

Conclusion: Our detailed morphometrics will be useful for planning of procedures in the posterior hard palate, including anesthetic infiltration of the greater palatine nerve, and avoidance of neurovascular bundle damage during periodontal surgery or palatal orthodontic implant placement.

## Introduction

Each side of the inferior surface of the palatine process of the maxilla has two longitudinal bony channels or palatal grooves (PGs) containing the greater palatine arteries and nerves, running parallel to the dental cementoalveolar margins at the level of the first and second molar teeth [1-4]. A central (middle) palatine spine (PS) separates these two PGs, and medial and lateral PSs may also be present [1-3]. The greater palatine artery and nerve occupy the lateral and medial PGs, respectively [2].

 There are now several accounts on the anatomy and morphometry of the bony PSs and their PGs [1-4]. There are also detailed descriptions of the anterior palatal mucosa between the canine and the first molar because it is uniformly thick and is widely used as an autogenous donor graft site in periodontal mucogingival surgery [2]. However, except for a 1991 article in Japanese by Ishinabe, we are not aware of any detailed descriptions and dimensions of mucosa covering the PSs and PGs in the posterior hard palate [5]. This knowledge would be clinically important for several reasons. First, bony canals encroaching on this mucosa have been described as occasionally bridging over the PGs from the PS margins, which may hinder attempts at needle infiltration of the nerve during local anesthesia [1]. Second, it could be important to avoid hemorrhagic complications from the artery during periodontal surgery or palatal orthodontic implant placement [3,6]. Third, the thickness and compressibility of this mucosa acts as a “foundation” for dentures in the posterior hard palate region [5]. Finally, just as tori palatinus and mandibularis may have overlying thin mucosa leading to sensitivity, inflammation, or ulceration, and the pterygoid hamulus may be implicated in similar stigmata of a pterygoid hamulus elongation syndrome, we conjectured that adequately thick mucosa would likely be a key factor in preventing the occurrence of a similar clinical scenario related to the PSs [7,8].

Using CT imaging, we rigorously evaluate the normative morphometric features of the PSs, PGs, and their overlying mucosa in a large number of patients.

## Materials and methods

Study approval

For this retrospective case-control study, we obtained ethical approval for this research study and waiver of individual patient consent from our local institutional review board administrative panel on human subjects in medical research. This study was conducted in accordance with the Helsinki Declaration of the ethical principles of medical research. 

Patient selection

Inclusion and Exclusion Criteria

**Figure 1:**
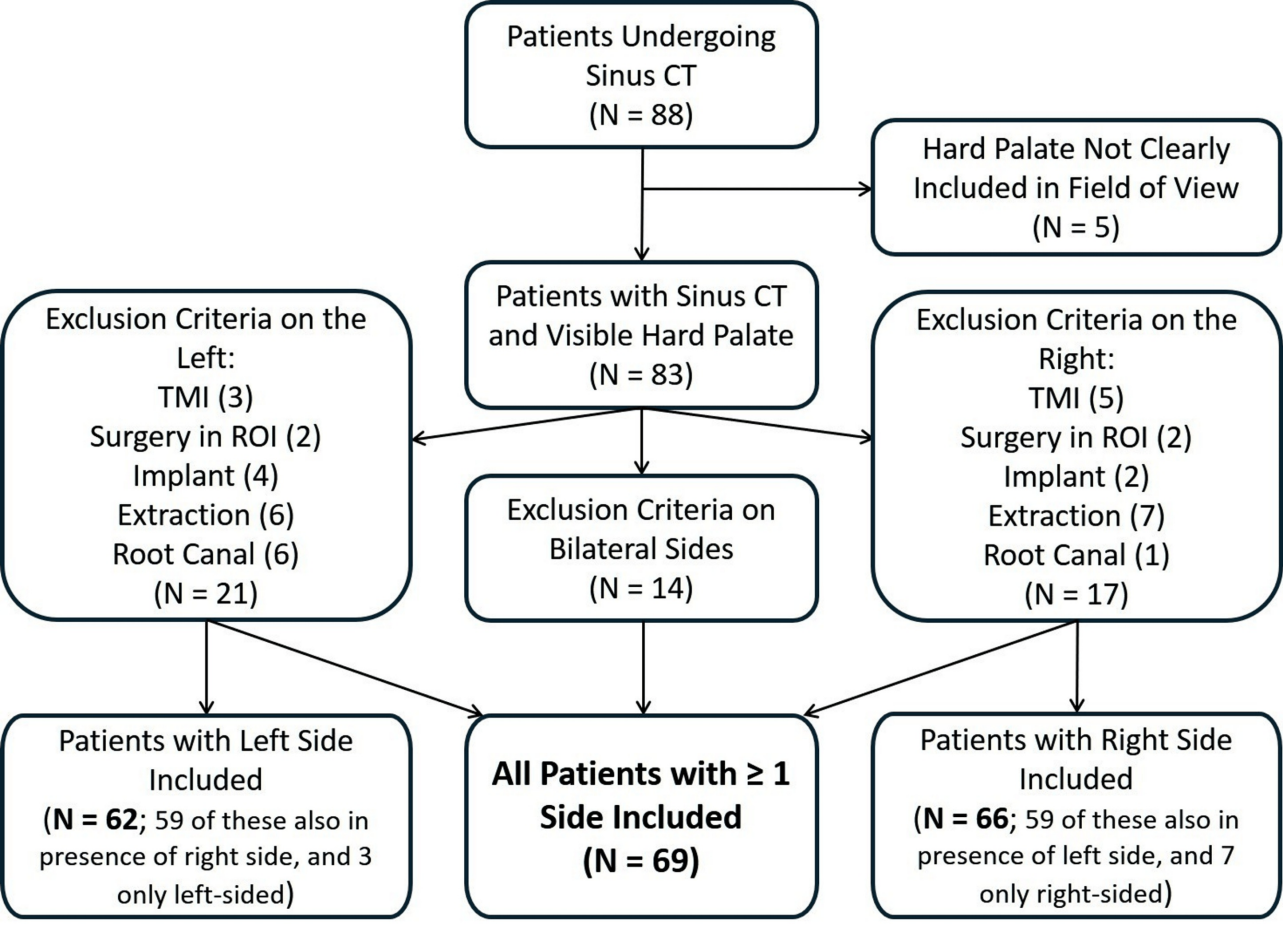
Patient selection Inclusion and exclusion criteria to an initial cohort of 88 patients undergoing CT imaging of the paranasal sinuses.

We applied inclusion and exclusion criteria to an initial cohort of 88 patients undergoing CT imaging of the paranasal sinuses (Figure 1) over a seven-week period from December 2024 to February 2025. These studies are usually planned to include a field-of-view with a lower limit just below the maxillary alveolar margin. We selected 69 patients with normal findings. The indications for CT scans in these selected patients were: chronic rhinosinusitis in 32, follow-up after endoscopic sinus surgery in six, nasal congestion in six, facial pain in six, nasal obstruction in five, polyposis in four, septal deviation or perforation in three, sinonasal mass in three, and other miscellaneous indications in four.

Imaging parameters

We reviewed coronal CT images that clearly depicted the hard palate, obtained using cone-beam CTs (acquisition at 90 kV and 5.0 mA, and a 250 μm voxel size); or conventional multidetector fan-beam CTs (acquisition at 120 kV and 125 mA, images reconstructed with 1 mm and 3 mm slice thickness using bone and soft tissue algorithms). Cone-beam CT is more appropriate for depicting bony structures and has been used to assess the PSs and PGs previously, but with appropriate windowing of images, the overlying soft tissue can be outlined easily [3,4,6]. Conventional CT has the advantage of providing separate soft tissue and bone kernels.

Image analysis

Two raters concurrently and collaboratively analyzed all anonymized images on a Sectra PACS review workstation (Linköping, Sweden). We used click-and-drag electronic calipers to obtain all morphometric measurements on digital images that optimally showed the PSs and tori palatinus in the coronal plane, both in the region of the posterior hard palate. There was an excellent reliability coefficient of > 0.90 for our first-time agreements as we jointly performed the objective measurements, and any dissimilarities were discussed and settled by additional consensus agreement.

Data collection

**Figure 2:**
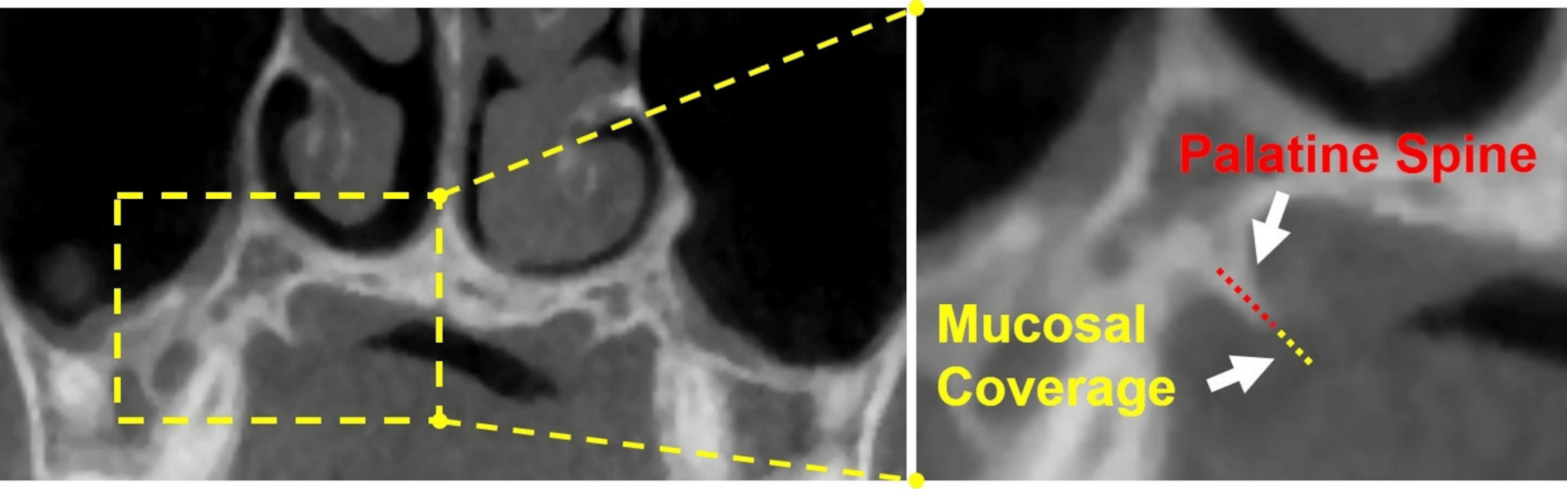
Location of PS and its mucosal coverage Left image shows a CT coronal image through the posterior hard palate demonstrating a right middle PS (within inset). Right image presents inset details of measurements made for the length of PS (red dotted line) and of its mucosal coverage (yellow dotted line). PS: Palatine spine

We recorded patient demographics and noted the presence of PSs and their shape (straight or curved), PGs, and tori palatinus; as well as laterality of findings, and location of PSs relative to the first and second molars. We then measured length of PSs from the hard palate margin, thickness of mucosa from the tip of each PS directly to the mucosal surface (mucosal coverage) (Figure 2), and height and width of tori palatinus. Lastly, in patients with no PSs, we measured mucosal coverage (in mm) at the angles formed by the hard palate and the right and left alveolar margins along similar trajectories as the dotted red plus yellow lines in Figure 2.

Statistical analysis

We performed statistical analyses using Python (version 3.12.2; Python Software Foundation, USA) using NumPy (v1.24.0), SciPy (v1.9.3), Seaborn (v0.11.2), and Matplotlib (v3.6.3). We tested the effect of independent variables (patient sex and PS side) on the measured dependent variables using independent samples t-tests and analysis of variance (ANOVA), as appropriate based on the number of groups being compared, and we performed linear regressions with age as the independent variable for each of the dependent variables, with significance set at p < 0.05.

## Results

**Figure 3:**
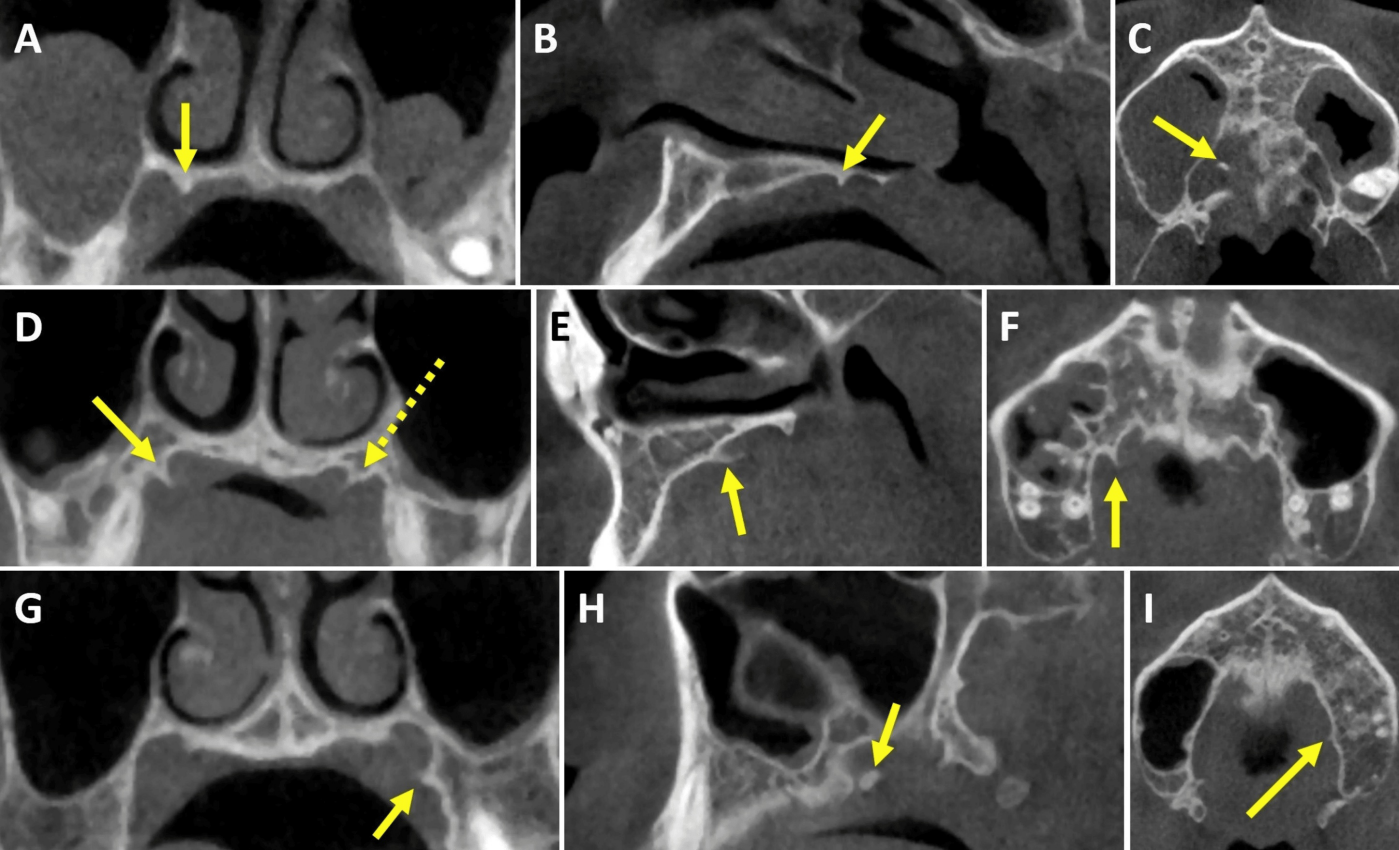
Multiplanar CT images showing the three locations for PSs Medial PSs are shown for the same patient in (A-C), middle PSs for a different patient in (D-F), and lateral PSs also for a different patient in (G-I). All arrows point to PSs in the coronal (A,D,G), sagittal (B,E,H), and axial planes (C,F,I). The dashed arrow in (D) shows a left middle PS in addition to the right middle PS (solid arrow). Coronal images were used for measurements of PSs and their mucosal coverage. Sagittal and axial images are shown here for clarification of PS anatomy and location. PS: Palatine spine

**Figure 4:**
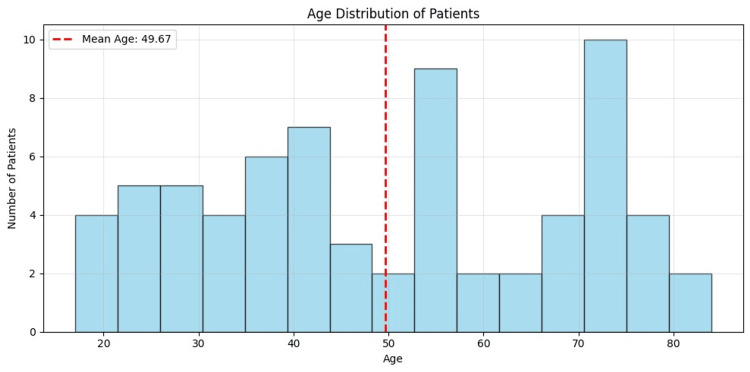
Age distribution of patients

**Figure 5:**
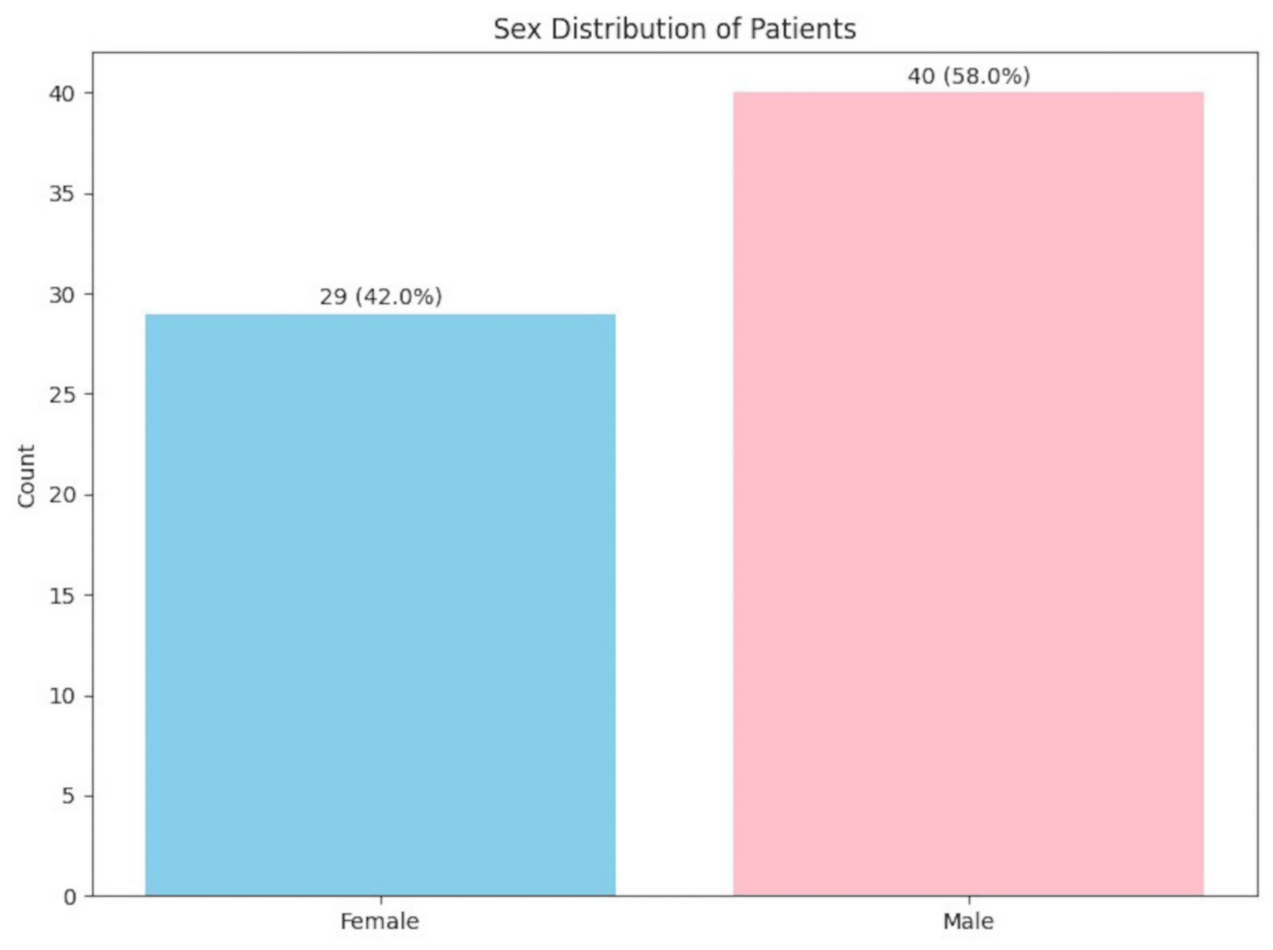
Sex distribution of patients

**Figure 6:**
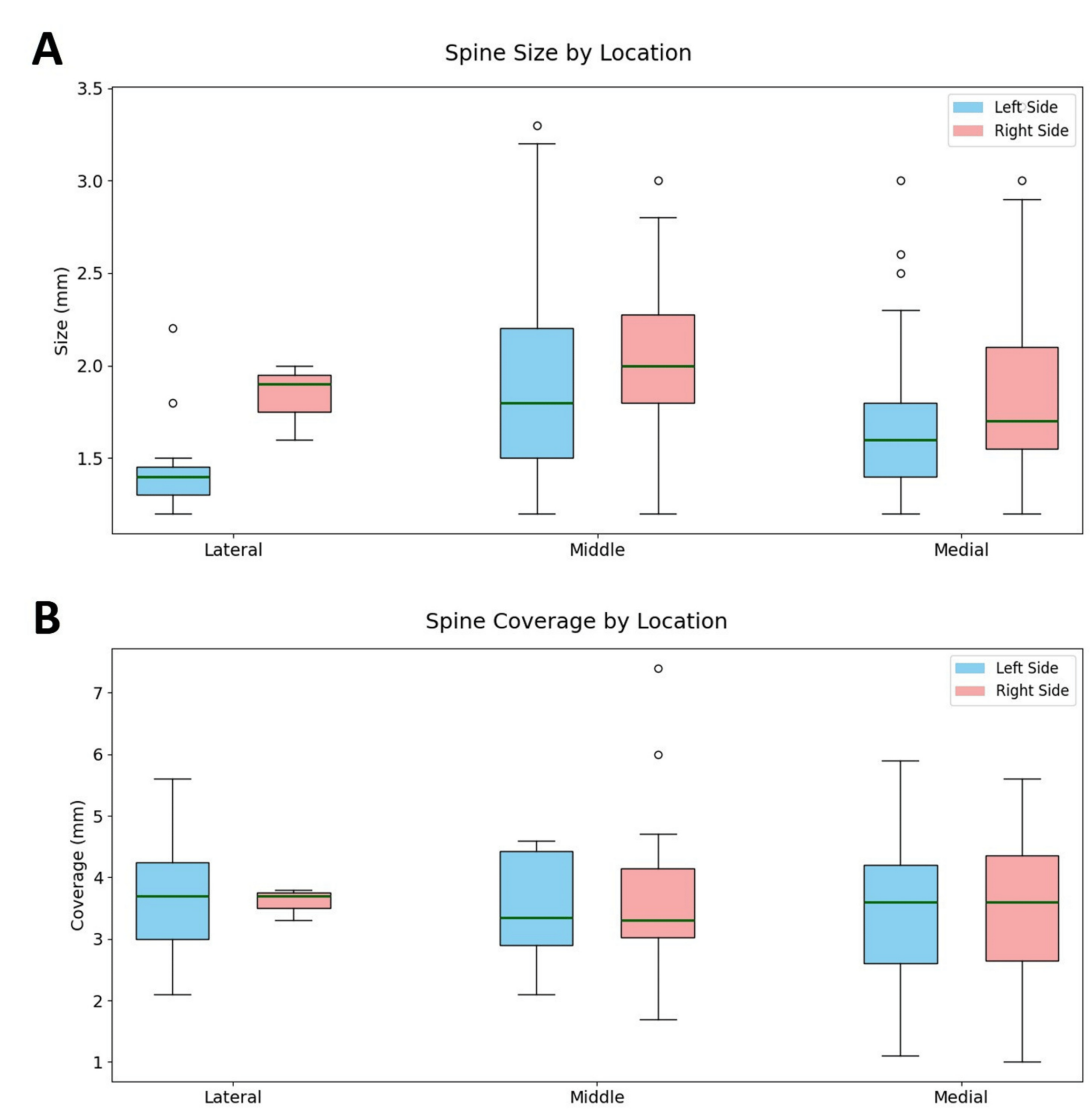
Distribution of lengths for PSs and their mucosal coverage by location and side (left and right) Box and whisker plots for (A) lengths (mm) of lateral/middle/medial PSs, and (B) thickness (mm) of mucosa covering the same PSs. Each box shows the middle 50% of values for each cohort, the median, and the minimum and maximum values that are not outliers. PS: Palatine spine

**Figure 7:**
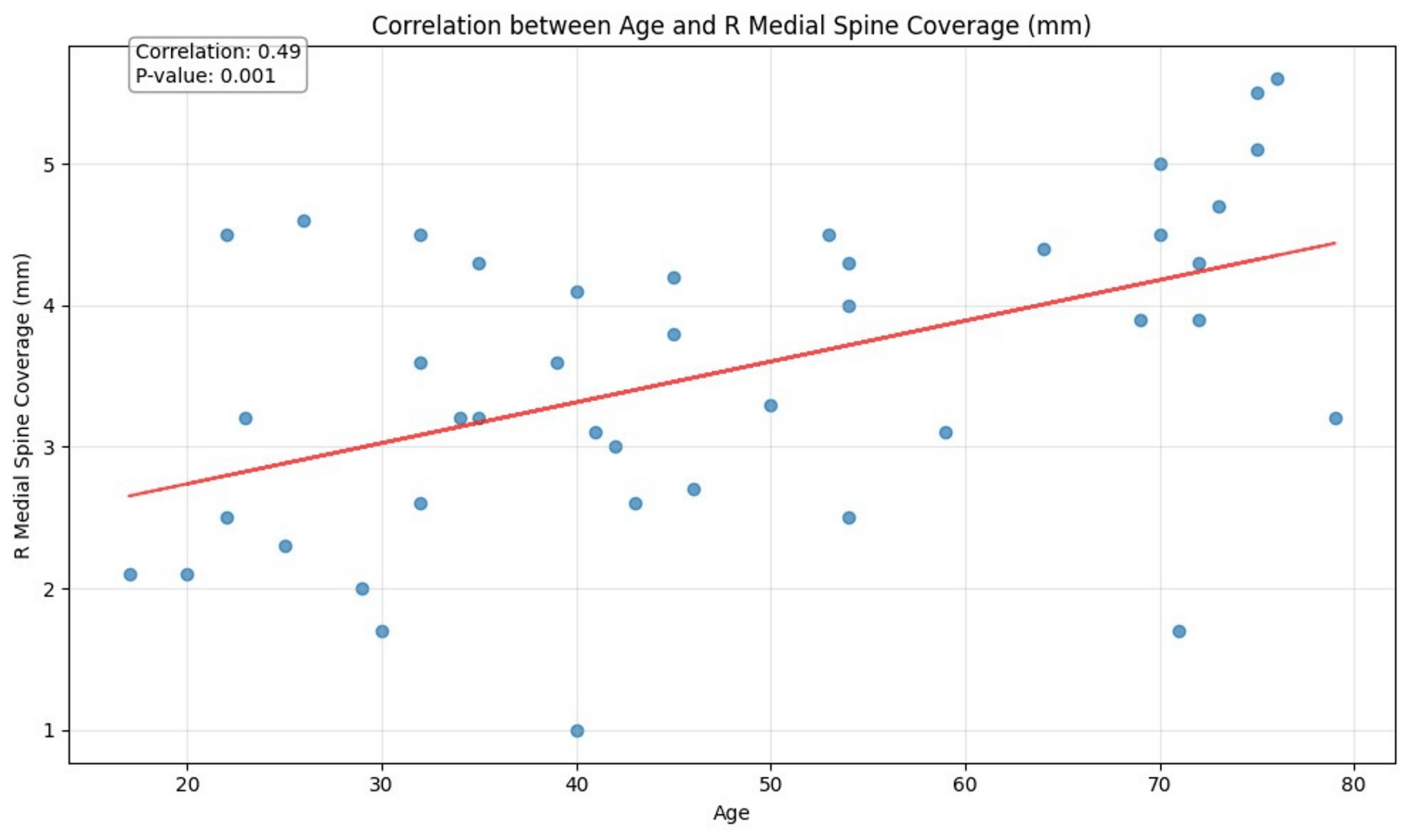
Correlation between age and right (R) medial spine coverage (mm)

**Figure 8:**
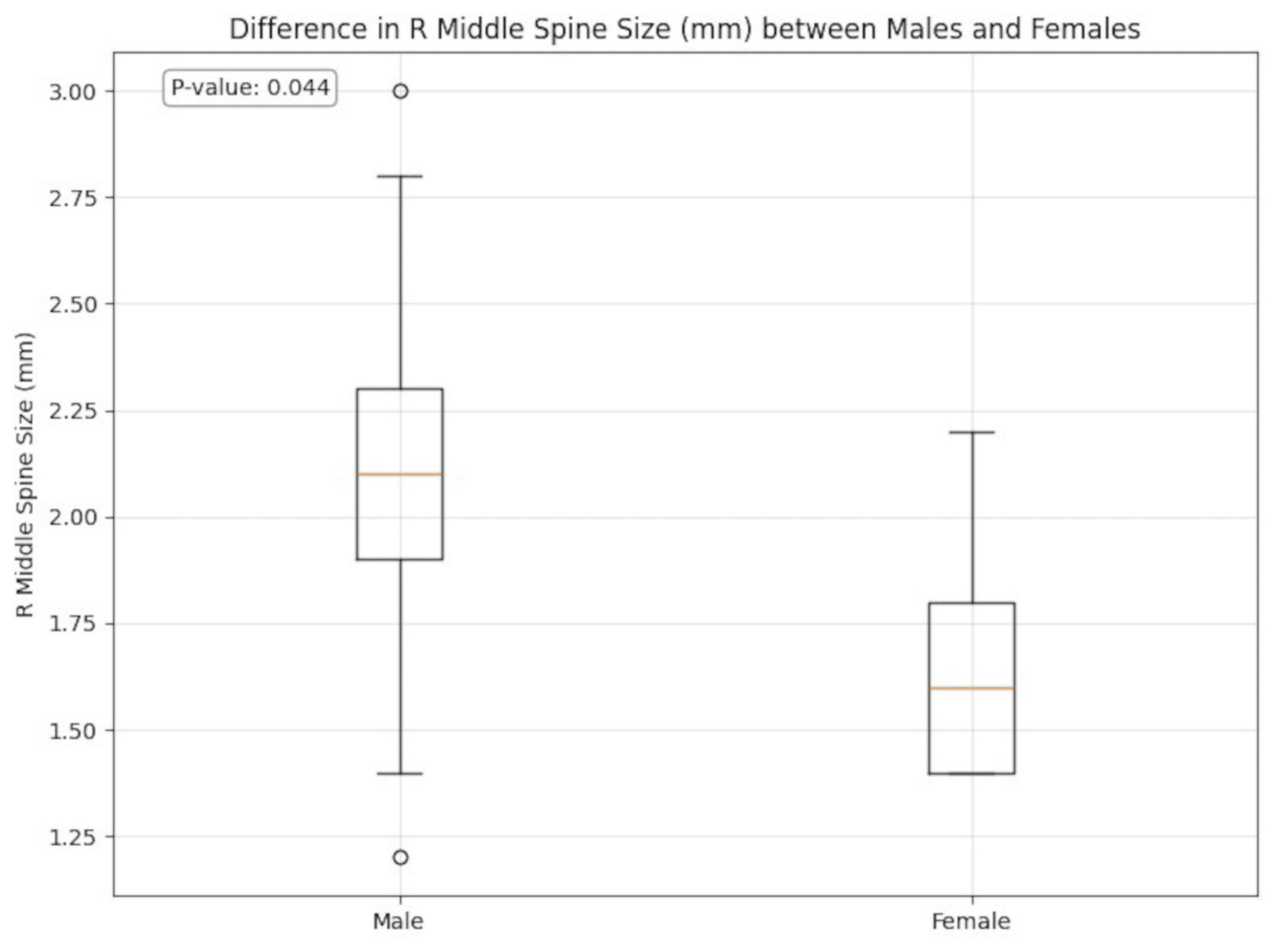
Difference in right (R) middle spine size (mm) between male and female subjects

**Figure 9:**
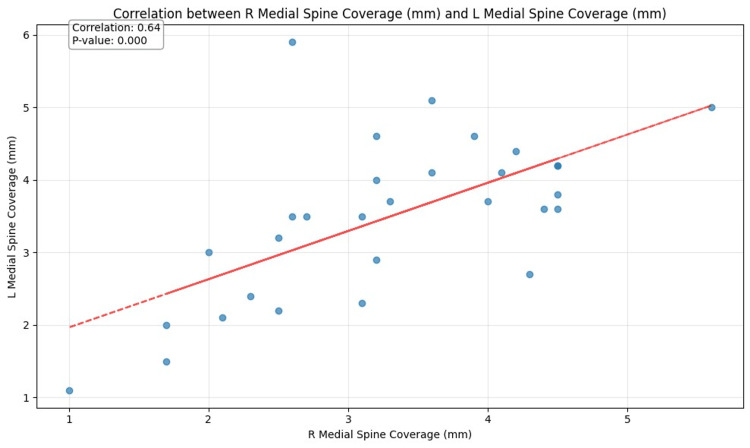
Correlation between right (R) medial spine coverage (mm) and left (L) medial spine coverage (mm)

On the selected 69 patients, we analyzed bilateral sides in 59 patients, the right side only in seven patients, and the left side only in three patients, for a total of 128 PSs (Figure 3) and their mucosal coverage (Figure 2). We obtained the following results: 1. distributions for patient age (Table 1 and Figure 4) and sex (Figure 5); 2. prevalence of noted parameters for PSs by side and location (Table 2), PS zones (Table 3), palatine grooves (Table 4), and PS shape (Table 5); 3. means and SDs for all measured parameters, including PS size and mucosal coverage (Table 6 and Figure 6), size of torus palatinus (Table 7), statistically significant correlations between PS characteristics and age (Table 8 and Figure 7), and sex (Table 9 and Figure 8), as well as between PS size and mucosal coverage (Table 10 and Figure 9).

**Table 1:** Age distribution analysis

Statistic	Value
Mean	49.67
Median	50.0
SD	19.40
Minimum	17.0
Maximum	84.0
Q1 (25%)	34.0
Q3 (75%)	70.0

**Table 2:** Prevalence of spines by side and location

Feature	Count	Percentage
Right Side Spine (Any Location)	54	78.3%
Left Side Spine (Any Location)	55	79.7%
R Lateral Spine	3	4.3%
R Middle Spine	22	31.9%
R Medial Spine	43	62.3%
L Lateral Spine	11	15.9%
L Middle Spine	16	23.2%
L Medial Spine	41	59.4%
R Lateral Shape - Straight	1	1.4%
R Lateral Shape - Straight	2	2.9%
R Middle Shape - Curved	7	10.1%
R Middle Shape - Straight	13	18.8%
R Middle Shape - Straight	2	2.9%
R Medial Shape - Curved	6	8.7%
R Medial Shape - Straight	1	1.4%
R Medial Shape - Straight	15	21.7%
R Medial Shape - Straight	20	29.0%
R Medial Shape - Straight	1	1.4%
L Lateral Shape - Straight	1	1.4%
L Lateral Shape - Straight	3	4.3%
L Lateral Shape - Straight	6	8.7%
L Lateral Shape - Straight	1	1.4%
L Middle Shape - Straight	9	13.0%
L Middle Shape - Straight	5	7.2%
L Middle Shape - Curved	2	2.9%
L Medial Shape - Curved	1	1.4%
L Medial Shape - Straight	2	2.9%
L Medial Shape - Straight	11	15.9%
L Medial Shape - Straight	26	37.7%
Right Bony Canal	0	0.0%
Left Bony Canal	0	0.0%
Taurus Palatines	32	46.4%

**Table 3:** Spine zone prevalence analysis For spine zone prevalence analysis, Zone 1 is PS adjacent to the first molar tooth. Zone 2 is PS adjacent to the second molar tooth. Zone 0 is PS adjacent to the second premolar tooth. 'No' indicates there were no PSs. PS: Palatine spine

R Spine Zone	Number	Percentage
1	48	71.64
0	6	8.96
No	13	19.40
L Spine Zone	Number	Percentage
1	45	68.18
2	3	4.55
No	11	16.67
0	7	10.61

**Table 4:** Grooves prevalence analysis

Feature	Count	Percentage
Right Side Grooves Presence	36	52.2%
Left Side Grooves Presence	39	56.5%

**Table 5:** Spine shape analysis

Location	Shape	Count	Percentage
R Lateral	Straight	2	66.7%
R Lateral	Straight	1	33.3%
R Middle	Straight	13	59.1%
R Middle	Curved	7	31.8%
R Middle	Straight	2	9.1%
R Medial	Straight	15	34.9%
R Medial	Straight	1	2.3%
R Medial	Curved	6	14.0%
R Medial	Straight	1	2.3%
R Medial	Straight	20	46.5%
L Lateral	Straight	6	54.5%
L Lateral	Straight	1	9.1%
L Lateral	Straight	3	27.3%
L Lateral	Straight	1	9.1%
L Middle	Straight	9	56.2%
L Middle	Curved	2	12.5%
L Middle	Straight	5	31.2%
L Medial	Straight	26	65.0%
L Medial	Straight	2	5.0%
L Medial	Straight	11	27.5%
L Medial	Curved	1	2.5%

**Table 6:** Spine size and coverage summary by location

Location	Measurement	Mean	SD	Median	Min	Max	Count
R Lateral	Size (mm)	1.83	0.21	1.9	1.6	2.0	3
R Lateral	Coverage (mm)	3.60	0.26	3.7	3.3	3.8	3
R Middle	Size (mm)	2.02	0.47	2.0	1.2	3.0	22
R Middle	Coverage (mm)	3.60	1.26	3.3	1.7	7.4	22
R Medial	Size (mm)	1.89	0.52	1.7	1.2	3.4	43
R Medial	Coverage (mm)	3.53	1.09	3.6	1.0	5.6	43
L Lateral	Size (mm)	1.45	0.30	1.4	1.2	2.2	11
L Lateral	Coverage (mm)	3.67	1.06	3.7	2.1	5.6	11
L Middle	Size (mm)	1.97	0.64	1.8	1.2	3.3	16
L Middle	Coverage (mm)	3.49	0.85	3.35	2.1	4.6	16
L Medial	Size (mm)	1.66	0.42	1.6	1.2	3.0	41
L Medial	Coverage (mm)	3.49	1.18	3.6	1.1	5.9	41

**Table 7:** Taurus palatinus size analysis

Statistic	Width (mm)	Length (mm)
Mean	8.3	2.42
Median	7.55	2.25
SD	2.05395	0.76
Minimum	4.8	1.3
Maximum	13.1	4.0

**Table 8:** Correlation between age and spine characteristics

Column	Correlation	P-value	N	Significance
R Lateral Spine Coverage (mm)	-0.411758	0.730	3	No
R Middle Spine Coverage (mm)	0.439718	0.041	22	Yes
R Medial Spine Coverage (mm)	0.491499	0.001	42	Yes
L Lateral Spine Coverage (mm)	0.265037	0.459	10	No
L Middle Spine Coverage (mm)	0.246205	0.358	16	No
L Medial Spine Coverage (mm)	0.251044	0.134	37	No
R Lateral Spine Size (mm)	-0.188982	0.879	3	No
R Middle Spine Size (mm)	-0.003452	0.988	22	No
R Medial Spine Size (mm)	0.040341	0.800	42	No
L Lateral Spine Size (mm)	0.21323	0.554	10	No
L Middle Spine Size (mm)	-0.29778	0.262	16	No
L Medial Spine Size (mm)	-0.174798	0.301	37	No
R Mucosal Coverage (mm) at Angle between Hard Palate and Alveolar Margin in Absence of a Spine	0.09676	0.790	10	No
L Mucosal Coverage (mm) at Angle between Hard Palate and Alveolar Margin in Absence of a Spine	0.531503	0.114	10	No

**Table 9:** Correlation between sex and spine characteristics

Column	Male Mean	Female Mean	P-value	Male N	Female N	Difference	Significance
R Middle Spine Coverage (mm)	3.71	3.26	0.510	17	5	0.445882	No
R Medial Spine Coverage (mm)	3.46	3.64	0.645	28	14	-0.178571	No
L Lateral Spine Coverage (mm)	3.53	3.62	0.893	4	6	-0.091667	No
L Middle Spine Coverage (mm)	3.71	3.02	0.207	11	5	0.689091	No
L Medial Spine Coverage (mm)	3.51	3.17	0.374	20	17	0.339412	No
R Middle Spine Size (mm)	2.12	1.68	0.044	17	5	0.437647	Yes
R Medial Spine Size (mm)	1.86	1.94	0.688	28	14	-0.071429	No
L Lateral Spine Size (mm)	1.43	1.50	0.713	4	6	-0.075	No
L Middle Spine Size (mm)	2.10	1.68	0.136	11	5	0.42	No
L Medial Spine Size (mm)	1.71	1.52	0.132	20	17	0.186471	No
R Mucosal Coverage (mm) at Angle between Hard Palate and Alveolar Margin in Absence of a Spine	6.97	7.16	0.892	3	7	-0.190476	No
L Mucosal Coverage (mm) at Angle between Hard Palate and Alveolar Margin in Absence of a Spine	7.85	6.58	0.143	6	4	1.275	No

**Table 10:** Correlation between mucosal coverage and spine size

Coverage Column	Other Column	Correlation	P-value	N	Significance
R Medial Spine Coverage (mm)	L Medial Spine Coverage (mm)	0.640599	0.000	30	Yes
L Middle Spine Coverage (mm)	R Middle Spine Size (mm)	0.775128	0.003	12	Yes
L Medial Spine Coverage (mm)	L Medial Spine Size (mm)	0.359311	0.021	41	Yes
R Medial Spine Coverage (mm)	R Middle Spine Coverage (mm)	0.557598	0.059	12	No
R Middle Spine Coverage (mm)	R Medial Spine Coverage (mm)	0.557598	0.059	12	No
L Middle Spine Coverage (mm)	L Medial Spine Coverage (mm)	0.851728	0.067	5	No
L Medial Spine Coverage (mm)	L Middle Spine Coverage (mm)	0.851728	0.067	5	No
R Middle Spine Coverage (mm)	L Middle Spine Coverage (mm)	0.504572	0.094	12	No
L Middle Spine Coverage (mm)	R Middle Spine Coverage (mm)	0.504572	0.094	12	No
R Middle Spine Coverage (mm)	L Medial Spine Size (mm)	0.571415	0.108	9	No
R Medial Spine Coverage (mm)	L Middle Spine Coverage (mm)	0.63914	0.122	7	No
L Middle Spine Coverage (mm)	R Medial Spine Coverage (mm)	0.63914	0.122	7	No
L Medial Spine Coverage (mm)	L Lateral Spine Size (mm)	-0.737388	0.155	5	No
L Lateral Spine Coverage (mm)	L Medial Spine Size (mm)	0.734107	0.158	5	No
R Middle Spine Coverage (mm)	R Middle Spine Size (mm)	0.292899	0.186	22	No
L Middle Spine Coverage (mm)	L Middle Spine Size (mm)	0.276085	0.301	16	No
R Medial Spine Coverage (mm)	R Medial Spine Size (mm)	0.136395	0.383	43	No
R Middle Spine Coverage (mm)	R Medial Spine Size (mm)	0.233811	0.465	12	No
L Medial Spine Coverage (mm)	L Middle Spine Size (mm)	0.344796	0.570	5	No
L Medial Spine Coverage (mm)	R Middle Spine Size (mm)	0.209024	0.589	9	No
L Lateral Spine Coverage (mm)	L Lateral Spine Size (mm)	0.17997	0.596	11	No
R Medial Spine Coverage (mm)	L Lateral Spine Size (mm)	0.297657	0.627	5	"No
L Medial Spine Coverage (mm)	R Medial Spine Size (mm)	0.079732	0.675	30	"No
R Medial Spine Coverage (mm)	L Lateral Spine Coverage (mm)	-0.257282	0.676	5	"No
L Lateral Spine Coverage (mm)	R Medial Spine Coverage (mm)	-0.257282	0.676	5	"No
R Medial Spine Coverage (mm)	R Middle Spine Size (mm)	0.134394	0.677	12	"No
L Lateral Spine Coverage (mm)	R Medial Spine Size (mm)	0.21682	0.726	5	"No
R Medial Spine Coverage (mm)	L Middle Spine Size (mm)	-0.136482	0.770	7	"No
L Middle Spine Coverage (mm)	R Medial Spine Size (mm)	0.127091	0.786	7	"No
R Medial Spine Coverage (mm)	L Medial Spine Size (mm)	0.037979	0.842	30	"No
L Middle Spine Coverage (mm)	L Medial Spine Size (mm)	0.101883	0.871	5	"No
R Middle Spine Coverage (mm)	L Middle Spine Size (mm)	0.047825	0.883	12	"No
L Medial Spine Coverage (mm)	L Lateral Spine Coverage (mm)	0.091086	0.884	5	"No
L Lateral Spine Coverage (mm)	L Medial Spine Coverage (mm)	0.091086	0.884	5	"No

Salient findings included patient mean age of 49.7 ± 19.4 years; 58% were men and 42% were women. PSs were almost equally prevalent on the right (in 78.3% of patients) and left (in 79.7%), with the right medial PS the most common (in 62.3%). Most PSs were straight in shape (in 53.3%). The right PGs were present in 52.2% and left in 56.5% of patients, but we did not observe bony canals over the PGs. A taurus palatinus was present in 46.6% of patients with a mean height of 2.4 ± 0.8 mm and width of 7.6 ± 2.3 mm. We observed a statistically significant sex difference for the right middle PS size, which is larger in men (p = 0.044).

Dimensions for mucosal coverage of all three PSs bilaterally varied in the range from 3.5 ± 0.9 mm to 3.7 ± 1.1 mm. We found several significant statistical correlations between mucosal coverage and other characteristics. The strongest of these statistical associations were: a positive correlation between age and right medial PS mucosal coverage (p = 0.001), and a positive correlation between right medial PS mucosal coverage and left medial PS mucosal coverage (p = 0.000). Notably, the right middle PS size was significantly correlated with its mucosal coverage (p = 0.003), and the left medial PS size was also significantly correlated with its mucosal coverage (p = 0.021).

## Discussion

There are three histological layers to the soft tissue coverage of the hard palate: a keratinized epithelium, a dense lamina propria, and a submucosa located below these two and containing Sharpey’s fibres that hold the lamina propria tightly to the periosteum [2]. In this study, we established comprehensive normative morphometrics for this soft tissue coverage of the PSs. We believe that the almost uniform dimensions we observed for mucosal coverage (mean 3.5 to 3.7 mm) over all three PSs bilaterally are more accurate than the mean 1.9 to 2.2 mm range recorded in 1991 using outdated B-mode ultrasound imaging because of the higher resolution of CT and its refined separation of bone edges from surrounding soft tissues [5].

Other notable findings of our study include the emergence of right PSs and their mucosal coverage in the most robust correlations with other recorded characteristics, including age and in males. We speculate that this may possibly be related to right-sided dominance of chewing in most people, but other contributing factors may also be at play [9].

Mansour and Huang have argued that a torus palatinus can alter the appearance of adjacent PGs and subsequently recorded a prevalence of 9% for tori palatinus among their patients [3]. Conversely, we recorded a prevalence of 46.4%, which is in line with several other studies [7]. Of note, we had included many instances of tiny (1-2 mm) elevations of cortical bone at the median palatine suture in our analysis, and these have been classified previously as small-sized tori palatinus (reviewed in García-García et al.) [7]. However, we found no correlations between torus palatinus size and other recorded features.

In agreement with Mansour and Huang but at odds with Hassanli and Mwaniki, we observed no bony canals that bridged PGs [3,10]. The neurovascular bundles in these cases are in direct contact with the soft tissue coverage of the hard palate [3].

There are several limitations to our study, including its retrospective nature, lack of direct visualization of the neurovascular bundle, and non-inclusion of pediatric patients to study the presence and development of PSs and PGs from childhood into adulthood. These will be subjects of future analyses.

## Conclusions

We report the normative dimensions for soft tissue coverage of the posterior hard palate overlying the PSs. The greater than 3 mm thick mucosa covering the PSs is unlikely to result in post-traumatic irritation and excessive keratinization, subsequent inflammation, or ulceration, as may be seen in other parts of the oral cavity. Our detailed morphometrics will be useful for planning of procedures in this region including anesthetic infiltration of the greater palatine nerve, and avoidance of neurovascular bundle damage during periodontal surgery or palatal orthodontic implant placement.
